# Hesperidin Attenuates Chronic Stress Induced Depressive‐Like Behavior in Mice: Pharmacological and Computational Evidence for PI3K Involvement

**DOI:** 10.1002/jbt.71176

**Published:** 2026-11-15

**Authors:** Mimansa Kandhwal, Amarjot Kaur Grewal, Komal Thapa, Varinder Singh, Manjinder Singh, Tanveer Singh, Thakur Gurjeet Singh, Sheikh F. Ahmad, Gamaleldin I. Harisa

**Affiliations:** ^1^ Chitkara College of Pharmacy Chitkara University Rajpura Punjab India; ^2^ Chandigarh Group of Colleges Jhanjeri, Mohali, Chandigarh Pharmacy College Jhanjeri Mohali Punjab India; ^3^ Department of Pharmaceutical Sciences and Technology Maharaja Ranjit Singh Punjab Technical University Bathinda Punjab India; ^4^ Department of Pharmaceutical Sciences and Natural products Central University of Punjab Bathinda Punjab India; ^5^ Department of Molecular and Integrative Physiology University of Illinois at Urbana‐Champaign Urbana Illinois USA; ^6^ Department of Pharmacology and Toxicology, College of Pharmacy King Saud University Riyadh Saudi Arabia; ^7^ Department of Pharmaceutics, College of Pharmacy King Saud University Riyadh Saudi Arabia

**Keywords:** chronic unpredictable mild stress, hesperidin, inflammation, oxidative stress, PI3K, Swiss albino mice

## Abstract

Depression is a multifactorial neuropsychiatric disorder associated with neuroendocrine, oxidative, inflammatory, and monoaminergic disturbances. The phosphoinositide 3‐kinase (PI3K) signaling pathway has emerged as an important regulator of neuronal survival and stress adaptation. The present study investigated the antidepressant‐like effects of hesperidin (HESP) and the possible involvement of PI3K signaling in chronic unpredictable mild stress (CUMS)‐induced depressive‐like behavior in mice. Animals were exposed to CUMS for 21 days and treated with HESP (100 and 200 mg/kg, orally), with LY294002 used to pharmacologically inhibit PI3K. HESP 200 mg/kg increased sucrose preference from approximately 36% to 67% and locomotor activity from approximately 75 to 140 crossings compared with the CUMS + vehicle group. HESP 200 mg/kg also reduced brain thiobarbituric acid reactive substances (TBARS) levels from approximately 19 to 12 nmol/mg protein and increased reduced glutathione (GSH) levels from approximately 14 to 24 µM/mg protein. Furthermore, corticosterone levels decreased from approximately 240 to 140 ng/mL, accompanied by improvements in inflammatory mediators and brain serotonin and dopamine levels. Pre‐administration of LY294002 markedly attenuated these beneficial effects of HESP. Molecular docking showed favorable HESP–PI3K interaction with a CHARMm‐based DOCKER (CDOCKER) interaction energy of −69.13 kcal/mol, while molecular dynamics simulation demonstrated complex stability over 100 ns. Collectively, the findings suggest that HESP exerts antidepressant‐like effects against chronic stress‐induced depressive pathology, possibly through modulation of PI3K signaling and its downstream regulation of neuroendocrine, oxidative, inflammatory, and monoaminergic pathways.

## Graphical abstract

Hesperidin protected against chronic stress‐induced behavioral, endocrine, oxidative, inflammatory, and monoaminergic alterations. Pharmacological inhibition and in silico analyses suggest that modulation of PI3K signaling may contribute to its antidepressant‐like and neuroprotective effects.

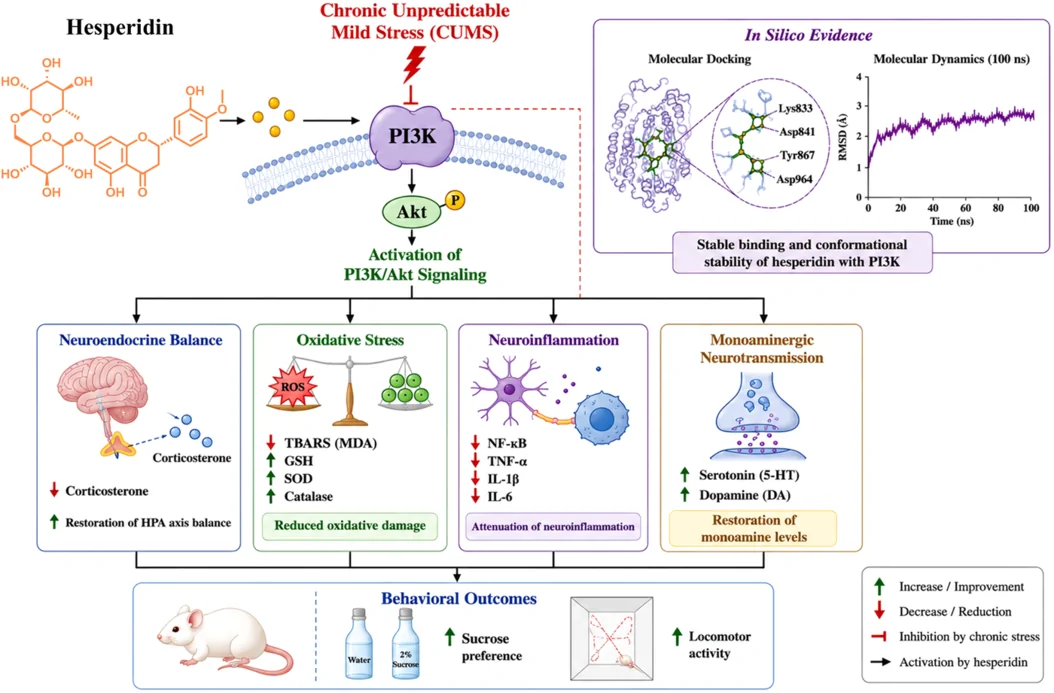

## 1 Introduction

Depression is a multifactorial neuropsychiatric disorder characterized by persistent disturbances in mood, motivation, cognition, and emotional behavior, and represents a major contributor to global disability and disease burden [1, 2, 3]. Despite the availability of several pharmacological treatments, including selective serotonin reuptake inhibitors (SSRIs; e.g., fluoxetine and sertraline), serotonin‐norepinephrine reuptake inhibitors (SNRIs; e.g., venlafaxine and duloxetine), tricyclic antidepressants (TCAs; e.g., amitriptyline and imipramine), monoamine oxidase inhibitors (MAOIs; e.g., phenelzine and tranylcypromine), and atypical antidepressants such as bupropion and mirtazapine, their clinical effectiveness remains limited by delayed therapeutic response, incomplete response or treatment resistance, relapse, and adverse effects. SSRIs and SNRIs are generally better tolerated but may cause gastrointestinal disturbances, sexual dysfunction, sleep disturbances, and withdrawal symptoms, whereas TCAs and MAOIs are associated with broader adverse‐effect profiles and clinically important safety and interaction concerns [4]. Such constraints highlight the need for finding effective and well‐tolerated novel therapeutic targets [5]. Chronic stress is considered one of the major etiological factors contributing to the onset and progression of depressive disorders. Persistent stress exposure can disturb hypothalamic‐pituitary‐adrenal (HPA) axis function, redox homeostasis, inflammatory signaling, and monoaminergic neurotransmission, thereby contributing to behavioral and neuronal dysfunction [6, 7, 8].

Over the past decade, intracellular signaling pathways have gained attention as they regulate neuronal survival, synaptic plasticity, inflammation, and emotional behavior. Amid signaling pathways, the phosphoinositide 3‐kinase (PI3K)/Akt signaling pathway has emerged as a crucial regulator of neuronal physiology [9]. PI3K signaling is involved in neurogenesis, neurotransmitter regulation, mitochondrial homeostasis, anti‐apoptotic responses, and maintenance of synaptic integrity. The phosphorylation of membrane phospholipids is catalyzed by PI3K, a lipid kinase that generates second messengers that activate downstream signaling molecules such as Akt. Akt activation promotes cell survival, inhibits apoptosis, and regulates cellular metabolism [10]. Beyond these established cellular functions, PI3K/Akt has attracted particular interest in depression because its activity can couple upstream neurotrophic and neurotransmitter signals to downstream changes in synaptic function and neuronal adaptation, processes that are disrupted by chronic stress [11]. Indeed, experimental studies have demonstrated reduced PI3K/Akt‐related signaling in stress‐induced depressive phenotypes, while pharmacological or molecular enhancement of this pathway produces antidepressant‐like responses [12]. More importantly, the functional relevance of PI3K has been demonstrated through pathway inhibition: blockade of PI3K with LY294002 has been shown to prevent or markedly attenuate the antidepressant‐like effects of several pharmacological interventions, including acetyl‐l‐carnitine, and AZD6765 [13, 14, 15]. These findings indicate that PI3K/Akt is not merely associated with depressive pathology but can participate in the biological response to antidepressant interventions, providing a pharmacologically testable mechanistic pathway. Alterations in PI3K‐mediated signaling have additionally been implicated in several neuropsychiatric and neurodegenerative disorders, suggesting PI3K as a promising therapeutic target for managing depression [16].

Natural bioactive compounds have gained considerable attraction as potential agents for the management of neuropsychiatric disorders because of their ability to target multiple pathological processes and better safety profiles [17, 18, 19]. Hesperidin (HESP), a flavanone glycoside, is abundant in citrus fruits and has gained interest due to its anti‐inflammatory, antioxidant, and neuroprotective benefits [20]. Studies suggest that HESP is beneficial for improving depressive‐like behaviors by reducing oxidative stress, suppressing inflammatory mediators, restoring monoaminergic neurotransmission, and modulating intracellular signaling cascades [21, 22, 23, 24]. Importantly, several reports indicate that HESP can regulate PI3K‐mediated signaling, thereby contributing to neuronal protection and functional recovery. However, the precise involvement of PI3K signaling in mediating the antidepressant‐like effects of HESP under chronic stress conditions remains inadequately explored. Therefore, the present study aimed to investigate the antidepressant‐like effects of hesperidin (HESP) and to determine the possible involvement of PI3K signaling in its protective effects against chronic unpredictable mild stress (CUMS)‐induced depressive‐like behavior. To achieve this aim, mice were subjected to a 21‐day CUMS paradigm and treated with HESP (100 and 200 mg/kg, p.o.), with fluoxetine included as a reference antidepressant. The antidepressant‐like effects of HESP were evaluated using the sucrose preference test (SPT) and open field test (OFT), while its effects on stress response, oxidative stress, neuroinflammation, and monoaminergic neurotransmission were assessed by measuring corticosterone, TBARS, GSH, SOD, catalase, NF‐κB, TNF‐α, IL‐1β, IL‐6, dopamine, and serotonin. The involvement of PI3K signaling was further examined pharmacologically using the PI3K inhibitor LY294002 in combination with HESP. In addition, molecular docking and 100‐ns molecular dynamics (MD) simulations were performed to explore the potential interaction and stability of the HESP‐PI3K complex.

## 2 Material and Methods

### 2.1 Chemicals

Hesperidin (HESP), 5,5′‐dithiobis‐(2‐nitrobenzoic acid) (DTNB), and 1,1,3,3‐tetraethoxypropane were purchased from Sigma‐Aldrich, USA. LY294002 was sourced from Merck Life Science Private Limited, Mumbai, India. Reduced glutathione (GSH) was procured from Loba Chemie, Pvt. Ltd. India. Enzyme‐linked immunosorbent assay (ELISA) kits used for estimation of inflammatory markers including nuclear factor‐kappa B (NF‐κB) (Kinesis Dx, India, Catalogue no. K02‐2879), interleukin‐1 beta (IL‐1β) (Krishgen Biosystems, Mumbai, India, Catalogue no. KB2083), interleukin‐6 (IL‐6) (Krishgen Biosystems, India, Catalogue no. KB2068), and tumor necrosis factor‐alpha (TNF‐α) (BT Labs, Shanghai, China, Catalogue no. E0117Mo) were obtained commercially. All other chemicals, solvents, and reagents used in the present study were of analytical grade.

### 2.2 Experimental Animals

Adult male Swiss albino mice weighing 25–30 g were used in the present investigation. The animals were procured from the Central Animal House Facility, Chitkara University, Rajpura, Punjab, India. Animals were housed in polypropylene cages containing clean paddy husk bedding, with three animals per cage, under standard laboratory environmental conditions, maintained at a temperature of 22°C ± 2°C with relative humidity of 55% ± 5% and a 12 h light/dark cycle (lights on from 07:00 to 19:00 h). Standard pellet diet and filtered drinking water were provided ad libitum throughout the experimental period. Animals were acclimatized to the laboratory environment for 1 week prior to initiation of the study to minimize stress‐related variability. All experimental procedures involving animals were conducted in accordance with the guidelines of the Committee for Control and Supervision of Experiments on Animals (CCSEA), Government of India. The experimental protocol was reviewed and approved by the Institutional Animal Ethics Committee (IAEC) under approval number IAEC/CCP/23/02/PR‐05. Every effort was made to minimize animal discomfort and distress throughout the experimental procedures. Animals were handled gently and routinely monitored during the study, and procedures involving terminal sample collection were performed under anaesthesia. All behavioral assessments were conducted during the light phase, between 09:00 and 13:00 h, under controlled and consistent environmental conditions. Animals were naïve to the respective behavioral tests and had no prior exposure to the corresponding test apparatus before behavioral assessment.

### 2.3 Experimental Design and Treatment Protocol

Animals were randomly divided into seven experimental groups, with eight animals in each group (*n* = 8). Group I (Naïve control) received vehicle treatment consisting of 0.5% carboxymethylcellulose (CMC) in distilled water and was not exposed to the CUMS protocol. Group II (CUMS control) was subjected to the CUMS protocol and received vehicle treatment orally once daily. Group III received fluoxetine (20 mg/kg, p.o.) along with CUMS exposure. Group IV and Group V received hesperidin (HESP) at doses of 100 mg/kg and 200 mg/kg (p.o.), respectively, during CUMS exposure. Group VI served as the LY294002 per se group and received LY294002 (10 mg/kg, i.p.) along with CUMS exposure. Group VII received LY294002 (10 mg/kg, i.p.) in combination with hesperidin (200 mg/kg, p.o.) during the stress protocol.

The doses of HESP (100 and 200 mg/kg, p.o.) were selected based on previously reported antidepressant‐like and neuroprotective effects of HESP in experimental models, including the dose range used in chronic stress‐induced depressive‐like behavior [25, 26]. LY294002 was administered at 10 mg/kg, i.p., as a pharmacological PI3K inhibitor dose previously used in vivo to investigate PI3K‐dependent mechanisms [27]. Hesperidin and fluoxetine were prepared as suspensions in 0.5% CMC and administered orally once daily. Drug treatments were administered 60 min prior to exposure to the daily stressor throughout the 21‐day experimental period [26]. Behavioral assessments, including the SPT and OFT, were performed following completion of the experimental schedule (Figure 1).

**Figure 1:**
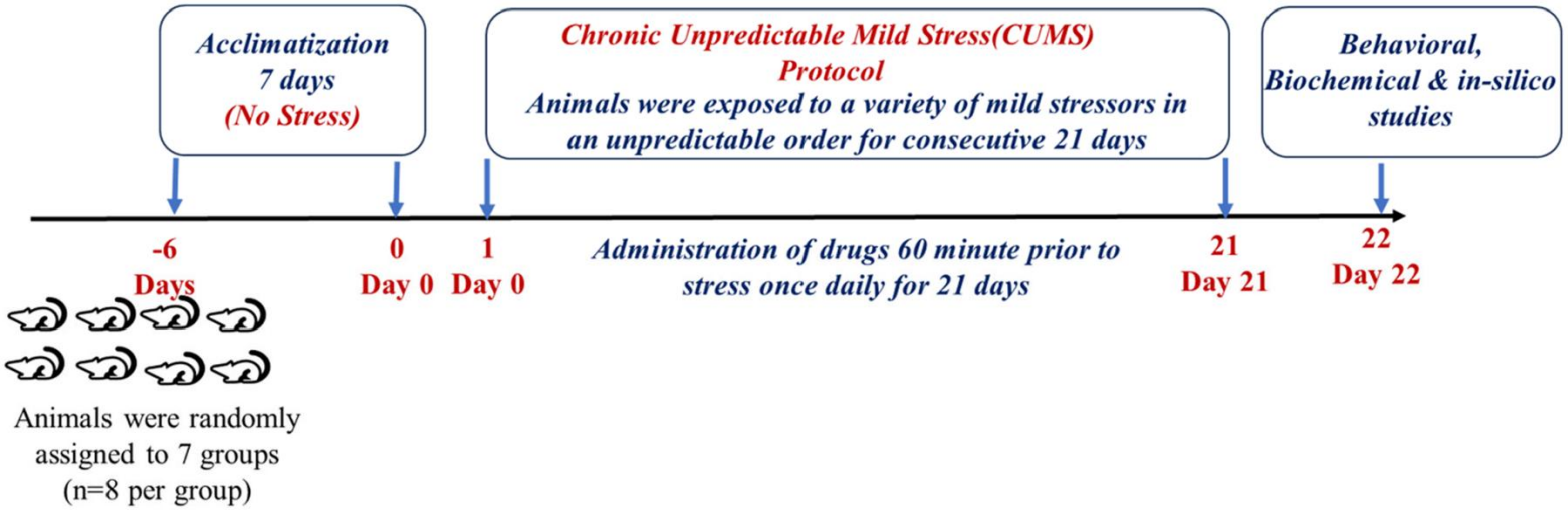
Experimental design and methodology.

### 2.4 CUMS Procedure

The CUMS model was employed to induce depressive‐like behavior in mice. The protocol was carried out for a duration of 21 consecutive days using a series of mild stressors presented in an unpredictable sequence to avoid stress adaptation [26, 28] (Figure 2). Animals assigned to the stress groups were exposed to different mild stressors as described in Figure 2. Stressors were applied randomly, and the same stressor was not repeated on consecutive days to maintain unpredictability throughout the experimental period. Only one stressor was administered at a time each day. Animals in the naïve control group were handled routinely but were not subjected to any stress procedures. Drug treatments were administered 60 min before stress exposure during the entire CUMS schedule. Following completion of the 21‐day stress paradigm, behavioral assessments were conducted to evaluate depressive‐like behavior in experimental animals.

**Figure 2:**
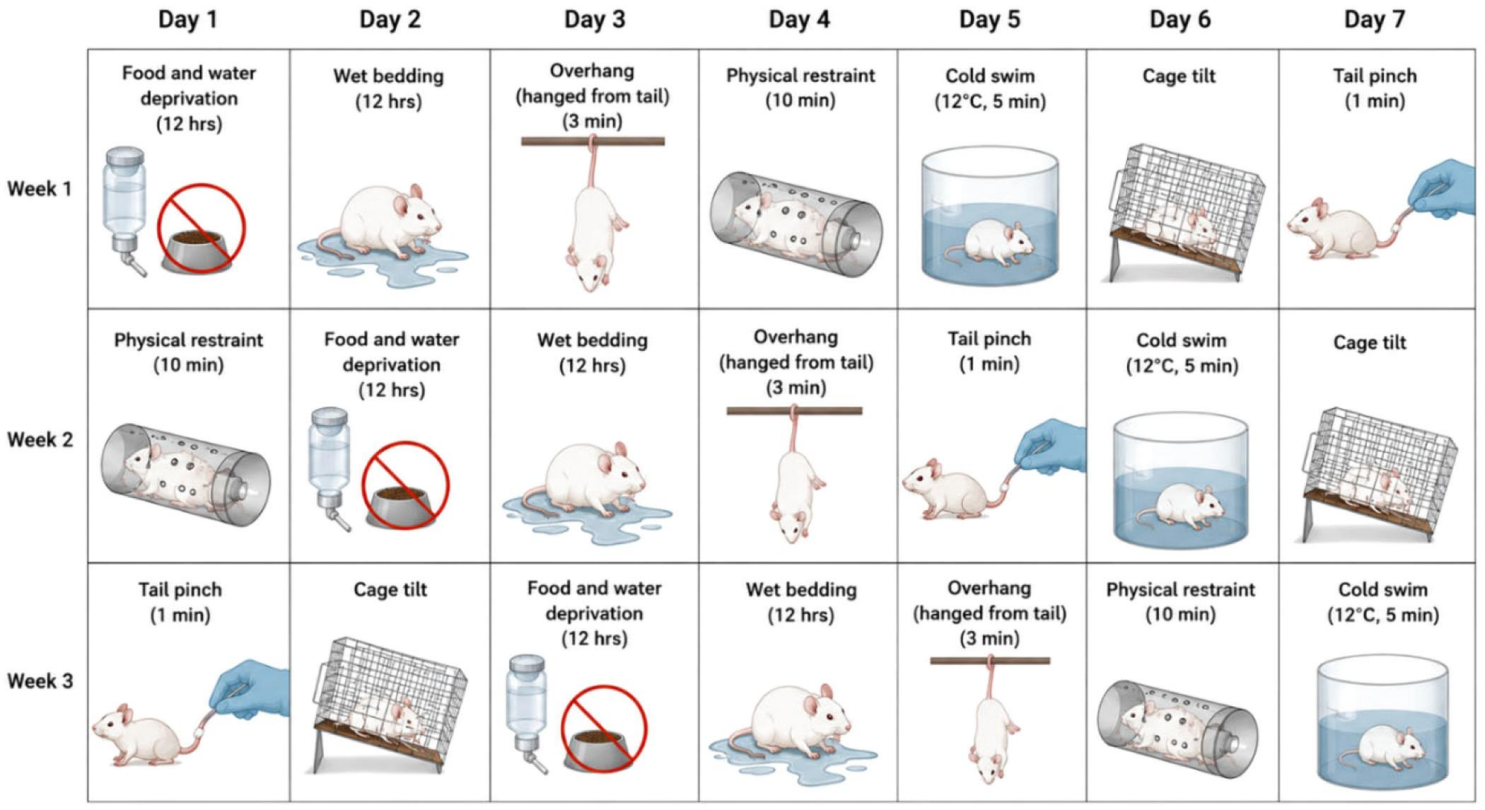
Schematic representation of the chronic unpredictable mild stress (CUMS) protocol employed for induction of depressive‐like behavior in mice over a period of 21 days.

### 2.5 Behavioral Studies

#### 2.5.1 SPT

SPT was performed using a two‐bottle choice paradigm to evaluate anhedonia‐like behavior in mice [28, 29, 30]. The test was conducted in two phases, namely the training phase and the testing phase.

#### 2.5.2 Training Phase

Prior to initiation of CUMS protocol, animals were habituated to the testing conditions. Mice were first exposed to two bottles containing regular drinking water for a period of 24 h. Following acclimatization, one of the bottles was replaced with 2% sucrose solution (w/v in water), while the second bottle contained plain drinking water. Animals were allowed free access to both bottles for the next 24 h.

After the adaptation period, animals were deprived of food and water for 24 h. Subsequently, mice were allowed to freely choose between the sucrose solution and drinking water for 1 h. To avoid positional bias, the positions of the bottles were alternated periodically. The intake of sucrose solution and water was measured by recording the weight of bottles before and after the test session.

Sucrose preference was calculated using the following formula:

Sucrose preference(%)=[Sucrose intake/(Sucrose intake+Water intake)]×100

#### 2.5.3 Testing Phase

On the 22nd day of the experimental protocol, approximately 24 h after exposure to the final stressor, animals were again subjected to the SPT. Mice were presented with two pre‐weighed bottles, one containing 2% sucrose solution and the other containing plain drinking water, for a duration of 1 h. The intake of both solutions was measured, and sucrose preference was calculated using the same formula as described for the training phase. A reduction in sucrose preference was considered indicative of anhedonia‐like behavior associated with depressive symptoms.

#### 2.5.4 OFT

OFT was performed to evaluate locomotion in experimental animals [29]. The apparatus consisted of a square wooden arena measuring 50 × 50 × 40 cm with high surrounding walls to prevent escape. The floor of the arena was divided into 25 equal squares (10 × 10 cm) marked with black lines. Each animal was individually placed in the center of the arena and allowed to explore freely for a duration of 5 min. The number of squares crossed by the animal with all four paws was recorded as an index of locomotor activity and exploratory behavior [31]. Increased exploratory movement was considered indicative of reduced depressive‐like behavior. After completion of each trial, the apparatus was cleaned thoroughly using 70% ethanol solution to eliminate olfactory cues and avoid interference between successive observations.

### 2.6 Biochemical Estimations

At the conclusion of behavioral testing, animals were anaesthetized using intraperitoneal injection of ketamine (80 mg/kg) combined with xylazine (10 mg/kg). Satisfactory anesthetic depth was confirmed by verifying complete abolition of the pedal withdrawal and corneal reflexes before proceeding further. Animals were thereafter euthanised by cervical dislocation. Whole blood was aspirated by cardiac puncture into plain collection tubes before brain removal. Serum was obtained following centrifugation at 3000 rpm for 5 min and preserved at −80°C for subsequent corticosterone measurement [32]. The whole brains were thereafter carefully removed, surface‐rinsed with chilled normal saline, suspended in chilled phosphate buffer (0.1 M, pH 7.4) at a concentration of 10% w/v, and mechanically disrupted using a tissue homogenizer [33]. Post‐centrifugation at 10,000 rpm for 15 min at 4°C, the recovered supernatant fraction served as the analytical source for determination of reduced glutathione (GSH), thiobarbituric acid reactive substances (TBARS), catalase, and superoxide dismutase (SOD) by the procedures of Beutler et al. [34], Ohkawa et al. [35], Hadwan et al. [36], and Kakkar et al. [37] respectively, consistent with methodologies reported previously from our group [33, 38, 39, 40]. Concentrations of NF‐κB, IL‐1β, IL‐6, and TNF‐α were measured from the same supernatant fraction using manufacturer‐validated commercial ELISA kits. A second brain portion was separately disrupted in 0.4 M perchloric acid, centrifuged identically, and the acid extract was used for spectrofluorimetric quantification of serotonin and dopamine as per Snyder et al. [41] and Guo et al. [42]. Protein estimation by Bradford assay [43] was performed on all supernatants, and biochemical values were normalized with respect to protein content.

### 2.7 Statistical Analysis

All data were expressed as mean ± standard deviation (SD). Statistical analysis was performed using GraphPad Prism 9.4.1 software (GraphPad Software, San Diego, CA, USA). The normality of data distribution was assessed using the Shapiro‐Wilk test, and all datasets satisfied the assumption of normality. As the data satisfied the assumptions of normality, differences among experimental groups were analyzed using one‐way analysis of variance (ANOVA), followed by Tukey's multiple‐comparison test. Following a significant ANOVA, all possible pairwise comparisons among the experimental groups were evaluated using Tukey's multiple‐comparison test. A value of *p* < 0.05 was considered statistically significant. All behavioral, biochemical, and statistical assessments were performed by investigators blinded to the treatment allocation.

### 2.8 In Silico Studies

#### 2.8.1 Molecular Docking Studies

Molecular docking studies were carried out using the CHARMm‐based docking tool (CDOCKER) implemented in Discovery Studio Client v20.1.0.19295 software (Dassault Systèmes BIOVIA, San Diego, CA, USA). The three‐dimensional crystal structure of PI3K with PDB ID: 3L54 was retrieved from the Protein Data Bank (PDB) [44, 45]. Protein preparation and optimization were performed in the DS Client workspace at pH 7.4 prior to docking analysis. The chemical structure of HESP was prepared and energy minimized before docking. The active binding site of PI3K was selected based on the co‐crystallized ligand and reported active site residues [26]. Docking simulations were performed using the CDOCKER protocol to evaluate ligand‐protein interactions and binding affinity. The docked conformations were analyzed based on ‐CDOCKER interaction energy and molecular interactions including hydrogen bonding, hydrophobic interactions, π‐alkyl interactions, and van der Waals forces [46].

#### 2.8.2 MD Simulation

MD simulations were performed for the docked HESP‐PI3K complex using the Desmond module of Schrödinger software. The stability and interaction profile of the protein‐ligand complex was analyzed using simulation interaction analysis over a simulation period of 100 ns [46]. Initially, an orthorhombic grid box was generated using the TIP3P solvable model system. The ionic strength of the system was adjusted using 0.15 M salt solution to maintain physiological conditions. System equilibration was carried out under continuous temperature conditions of 310 K using the Nose‐Hoover chain thermostat, while a constant pressure of 1.01325 bar was maintained using the Martyna‐Tobias‐Klein barostat method [47]. Following the 100 ns trajectory, the structural stability and dynamic fluctuations of the protein‐ligand complex were quantitatively assessed by analyzing the Root Mean Square Deviation (RMSD) and Root Mean Square Fluctuation (RMSF) of the system over time.

## 3 Results

### 3.1 Effect of Hesp on the SPT

Sucrose preference remained comparable across all groups during the baseline phase, with no significant differences observed, confirming uniform hedonic status before stress exposure (Figure 3A). Following CUMS induction, a marked reduction in sucrose preference was observed in the CUMS + vehicle group compared with naïve animals, indicating the development of anhedonia. Intervention with HESP significantly mitigated this deficit. Both treatment groups exhibited a restoration of sucrose preference compared to CUMS + vehicle. The observed effects were comparable to those seen with fluoxetine, indicating robust antidepressant‐like activity. Administration of LY294002 alone did not significantly influence sucrose preference compared to the CUMS control group. However, when combined with HESP, the beneficial effect of HESP 200 was significantly blunted (*p* < 0.0001 vs. HESP 200), resulting in reduced sucrose preference approaching CUMS levels.

**Figure 3:**
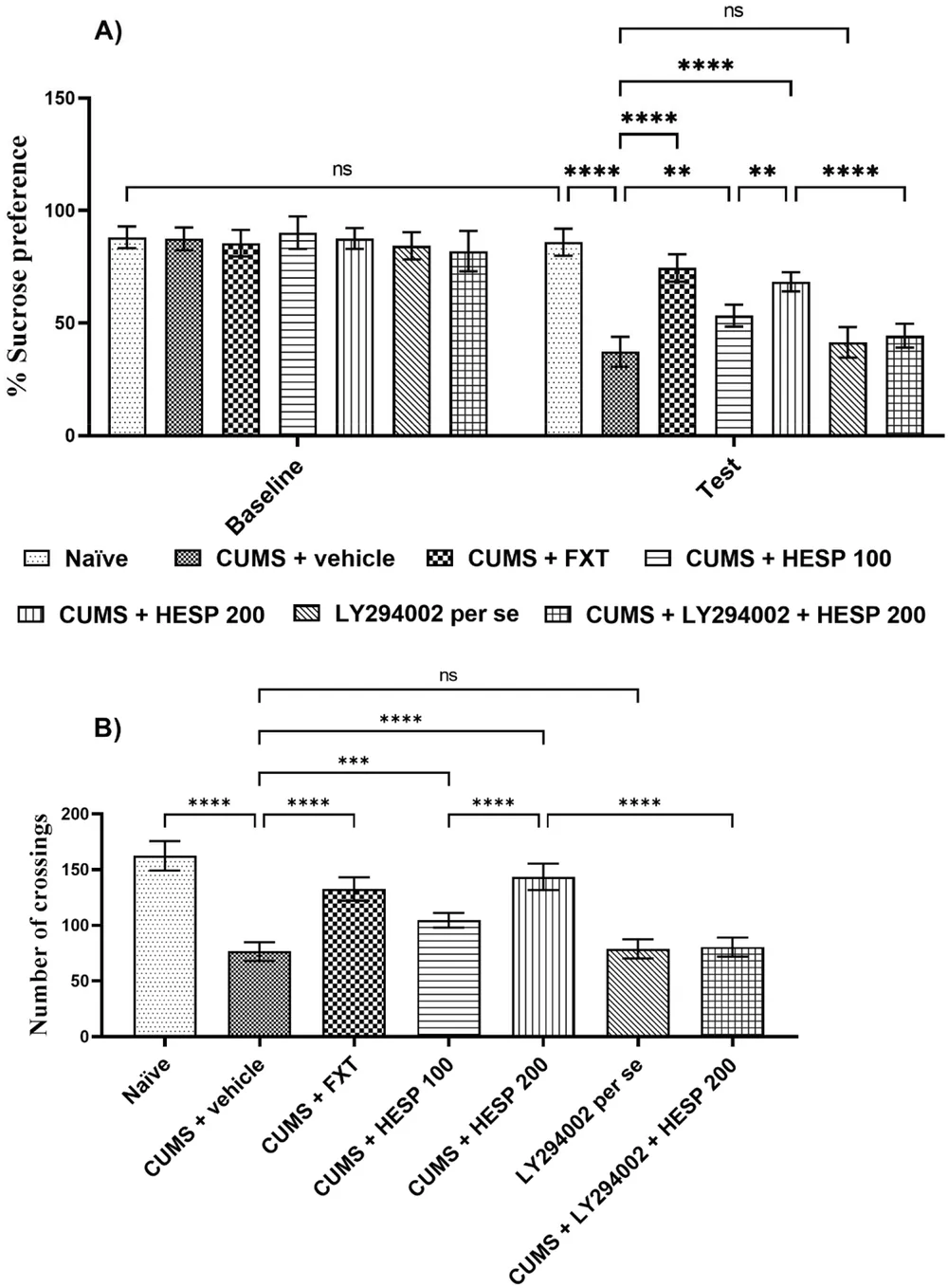
Effect of HESP on (A) sucrose consumption in the Sucrose preference test and (B) number of crossing in the Open Field Test of animals exposed to CUMS. Values are presented as mean ± SD (*n* = 8) and analyzed by one‐way ANOVA followed by Tukey's multiple comparison test. ****p < 0.0001, ***p < 0.001, **p < 0.01, ns‐ non‐significant. CUMS, chronic unpredictable mild stress; FXT, fluoxetine; HESP, hesperidin; LY294002, phosphoinositide 3‐kinase inhibitor; SD, standard deviation; ns, non‐significant.

### 3.2 Effect of HESP on the Number of Crossings in the OFT

The locomotor activity was considerably suppressed in chronic stress exposure, as observable by a remarkable reduction in the number of crossings in the CUMS + vehicle group compared to naïve animals, depicting behavioral impairment associated with depressive‐like states (Figure 3B). Pharmacological treatments reversed the impairment to varying degrees. Fluoxetine‐treated animals revealed a significant increase in crossings relative to the CUMS group. A similar trend was observed with HESP, where both 100 and 200 mg/kg doses enhanced locomotor activity compared to CUMS + vehicle. Administration of LY294002 alone failed to improve locomotor performance and remained comparable to the CUMS control. The effect of HESP (200 mg/kg) was notably reduced by co‐treatment with LY294002, as seen by a considerable drop in crossings when compared to the HESP‐alone group.

### 3.3 Effect of HESP on Oxidative Stress Markers in CUMS‐Induced Mice

CUMS causes a clear redox imbalance, as revealed by a significant increase in lipid peroxidation assessed by raised levels of TBARS along with a marked drop in endogenous antioxidant systems, including reduced glutathione (GSH), SOD, and catalase activities, in the CUMS + vehicle group compared to naïve animals (Figure 4). Pharmacological treatments differentially mitigated these alterations. Fluoxetine markedly reduced TBARS levels while restoring GSH content and enzymatic antioxidant activities (SOD and catalase) toward baseline values. HESP treatment also significantly improved oxidative imbalance, demonstrating a consistent protective profile across all markers. Both 100 and 200 mg/kg doses attenuated lipid peroxidation and enhanced antioxidant defenses, evident as lower TBARS and greater recovery of GSH, SOD, and catalase levels as compared to CUMS + vehicle. However, oxidative disturbances could not be addressed by LY294002 treatment alone; TBARS remained increased, and antioxidant markers constantly declined, just like in the CUMS group (Figure 4). Importantly, LY294002 co‐administration with HESP (200 mg/kg) remarkably diminished the therapeutic benefits of HESP across all measured parameters, as reflected by increased TBARS levels and decreased GSH, SOD, and catalase activities compared to HESP treatment alone, reverting values toward those observed under stress conditions.

**Figure 4:**
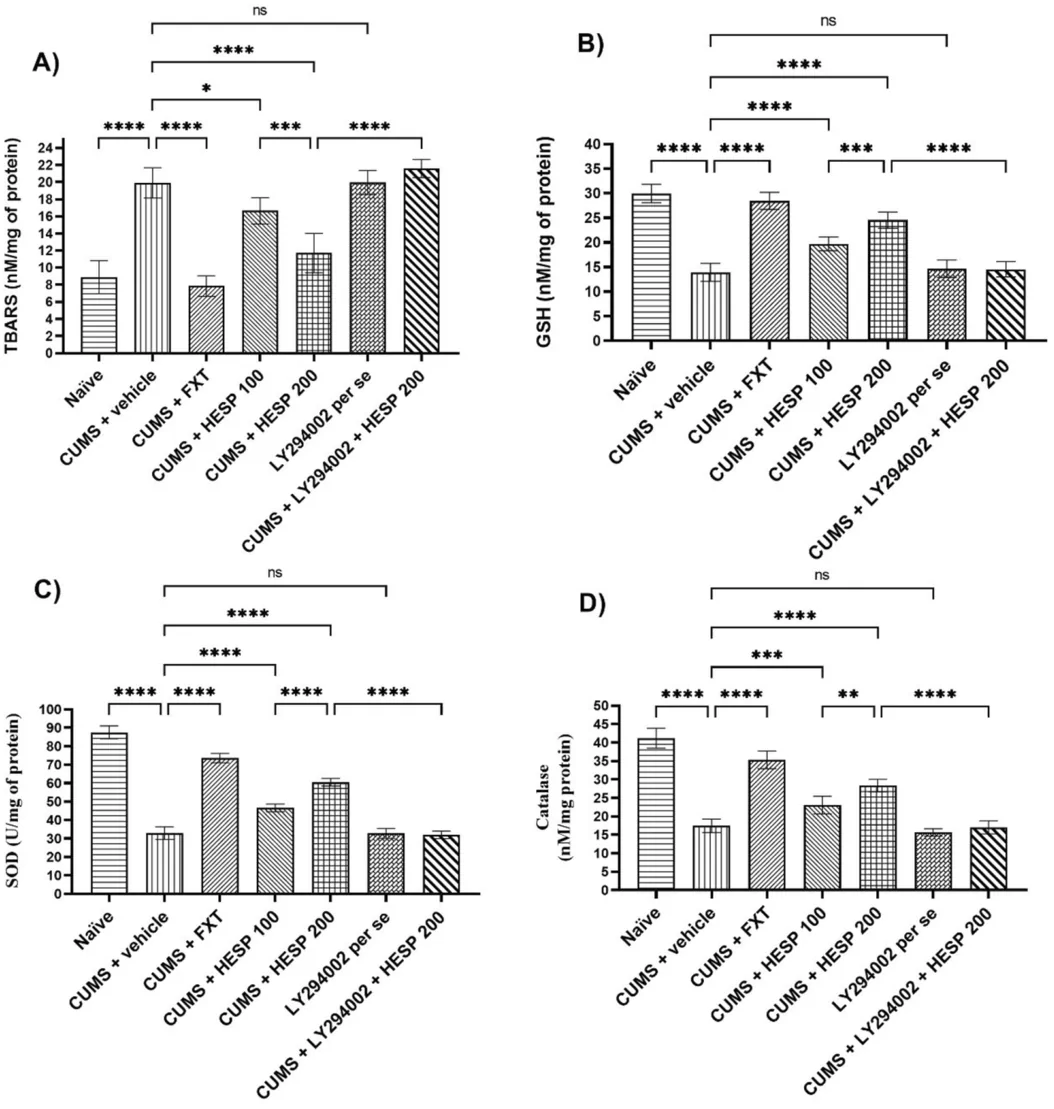
Effect of HESP on brain (A) TBARS, (B) GSH, (C) SOD and (D) Catalase levels of animals exposed to CUMS. Values are presented as mean ± SD (*n* = 8) and analyzed by one‐way ANOVA followed by Tukey's multiple comparison test. ****p < 0.0001, ***p < 0.001, **p < 0.01, *p < 0.05. CUMS, chronic unpredictable mild stress; FXT, fluoxetine; GSH, reduced glutathione; HESP, hesperidin; FXT, fluoxetine; LY294002, phosphoinositide 3‐kinase inhibitor; ns, non‐significant; SD, standard deviation; SOD, superoxide dismutase; TBARS, thiobarbituric acid reactive substances.

### 3.4 Effect of HESP on Neuroinflammatory Markers in CUMS‐Induced Mice

Marked increase in the levels of proinflammatory mediators such as TNF‐α, IL‐6, IL‐1β, and NF‐κB levels was observed in the CUMS + vehicle group compared to naïve animals, which confirmed the robust activation of inflammatory pathways under chronic stress (Figure 5). Pharmacological treatments differentially modulated this response. Fluoxetine administration significantly reduced all assessed inflammatory mediators, including NF‐κB and cytokines, compared to the CUMS group. Similar to CUMS + vehicle, HESP significantly reduced the levels of TNF‐α, IL‐6, IL‐1β, and NF‐κB at both 100 and 200 mg/kg.

**Figure 5:**
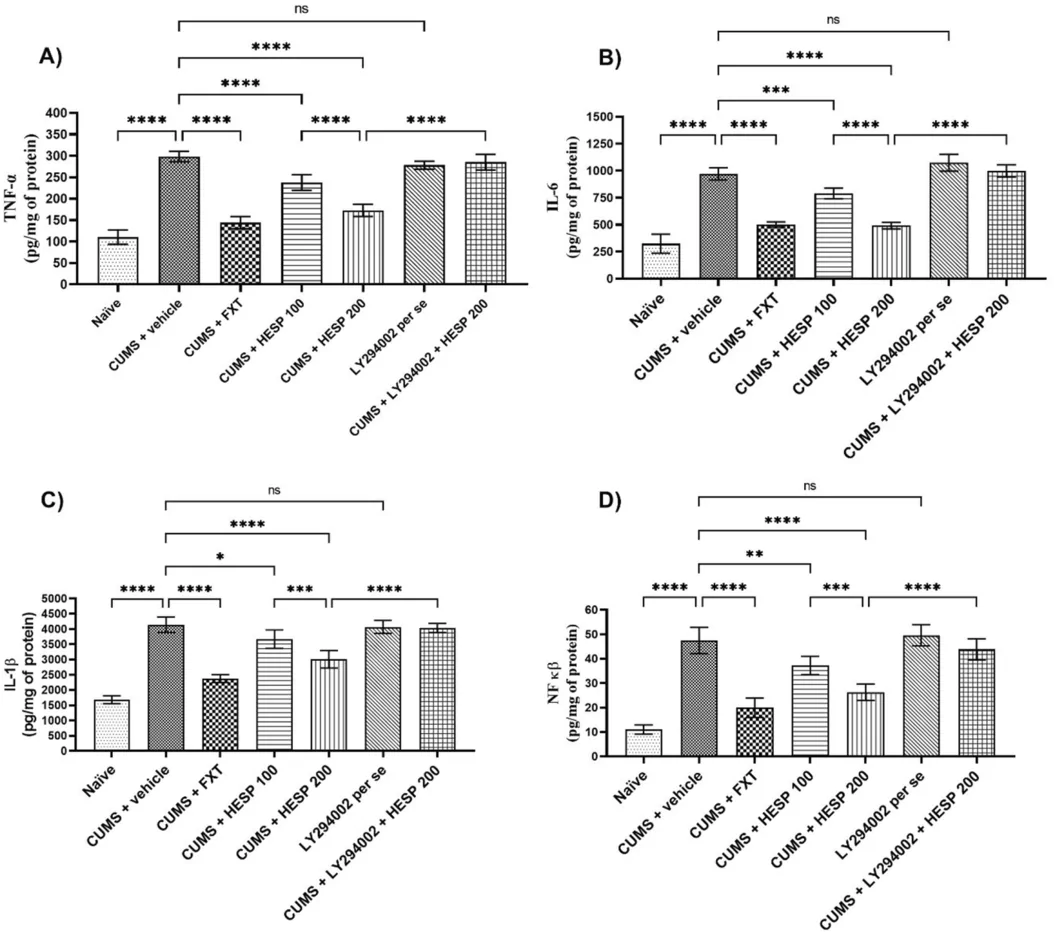
Effect of HESP on brain (A) TNF‐α; (B) IL‐6; (C) IL‐1β and (D) NF‐κB levels of animals exposed to CUMS. Values are presented as mean ± SD (*n* = 8) and analyzed by one‐way ANOVA followed by Tukey's multiple comparison test. ****p < 0.0001, ***p < 0.001, **p < 0.01, *p < 0.05. CUMS, chronic unpredictable mild stress; FXT, fluoxetine; HESP, hesperidin; IL‐6, interleukin‐6; IL‐1β, interleukin‐1 beta; LY294002, phosphoinositide 3‐kinase inhibitor; NF‐κB, nuclear factor‐kappa B; ns, non‐significant; SD, standard deviation; TNF‐α, tumor necrosis factor‐alpha.

Conversely, there was no discernible anti‐inflammatory effect from LY294002 alone; cytokine and NF‐κB levels remained high and similar to the CUMS control. Furthermore, the anti‐inflammatory effects of HESP (200 mg/kg) were effectively counteracted by co‐treatment with LY294002, as demonstrated by a notable increase in TNF‐α, IL‐1β, IL‐6, and NF‐κB levels in comparison to HESP treatment alone, restoring the inflammatory profile to that of stressed animals.

### 3.5 Effect of HESP on Brain Neurotransmitters

Chronic stress distinctly impaired monoaminergic signaling, as reflected by significant reductions in both dopamine and serotonin levels in the CUMS + vehicle group compared to naïve animals, indicating disruption of key neurotransmitter systems associated with mood regulation (Figure 6A,B). Fluoxetine markedly elevated both dopamine and serotonin levels relative to stressed controls, consistent with its known monoaminergic action. HESP treatment also improved neurotransmitter levels across both systems. In contrast, LY294002 alone failed to reverse monoaminergic depletion, as the levels of both dopamine and serotonin remained similar to CUMS group. Notably, inhibition of PI3K/Akt signaling significantly compromised the efficacy of HESP, as co‐treatment with LY294002 attenuated the recovery of both neurotransmitters, resulting in levels that reverted toward those seen under chronic stress conditions.

**Figure 6:**
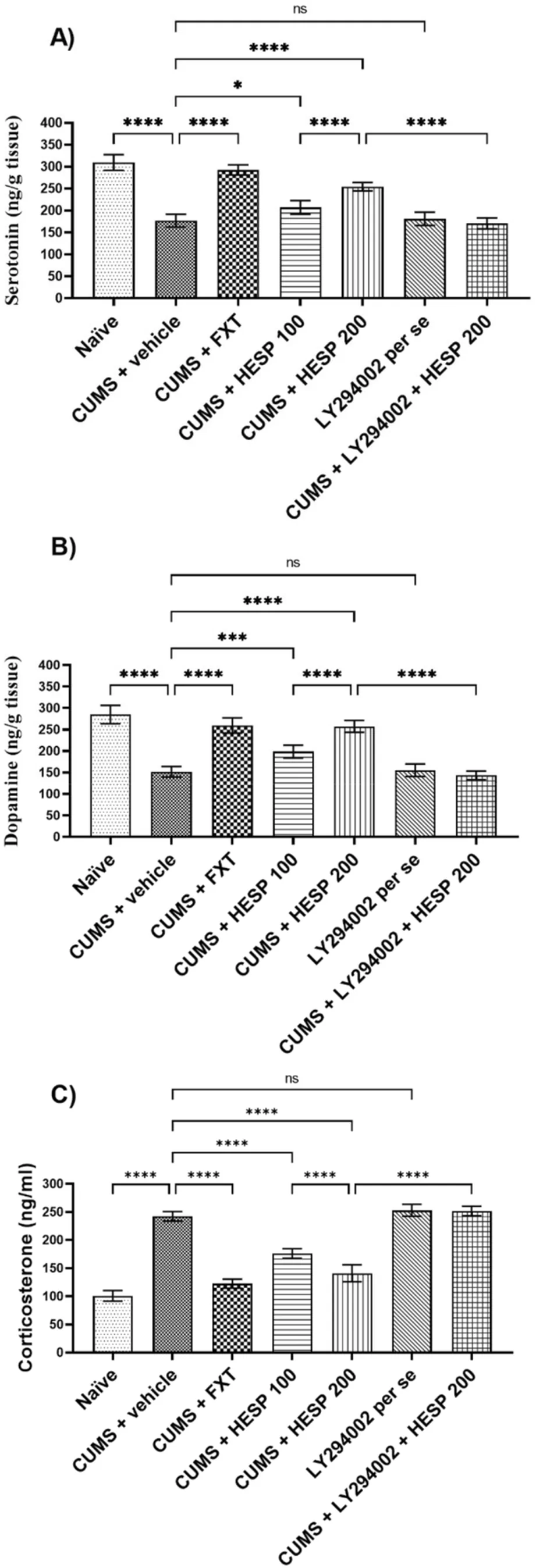
Effect of HESP on brain (A) Serotonin, (B) Dopamine and (C) Corticosterone levels in CUMS‐induced mice. Values are presented as mean ± SD (*n* = 8) and analyzed by one‐way ANOVA followed by Tukey's multiple comparison test. ****p < 0.0001, ***p < 0.001, *p < 0.05. CUMS, chronic unpredictable mild stress; FXT, fluoxetine; HESP, hesperidin; LY294002, phosphoinositide 3‐kinase inhibitor; ns, non‐significant; SD, standard deviation.

### 3.6 Effect of HESP on Corticosterone Levels

A clear hyperactivation of the stress axis was evident following CUMS exposure, as indicated by a marked elevation in corticosterone levels in the CUMS + vehicle group compared to naïve animals (Figure 6C). Intervention with fluoxetine significantly reduced circulating corticosterone compared to stressed animals, bringing levels closer to baseline. HESP also moderated this stress response, with both 100 and 200 mg/kg doses producing significant reductions in contrast to CUMS + vehicle group. In contrast, LY294002 alone did not attenuate corticosterone elevation and remained comparable to the CUMS group. Furthermore, co‐administration of LY294002 with HESP (200 mg/kg) abolished the corticosterone‐lowering effect of HESP, as reflected by significantly higher levels compared to HESP treatment alone, approaching those observed in stressed controls.

### 3.7 Molecular Docking and Dynamics Analysis of HESP With PI3K

Docking studies revealed that HESP binds favorably within the catalytic pocket of PI3K (PDB ID: 3L54), exhibiting strong interaction energies (−64.36 kcal/mol in docking; −CDOCKER interaction energy of 69.1339) (Table 1). The ligand was well accommodated within the active site and formed multiple stabilizing interactions with residues critical for enzymatic function (Figure 7). At the binding interface, HESP established conventional hydrogen bonds with key residues including Asp841, Lys833, Tyr867, Asp964, and Val882, which are essential for ligand anchoring and orientation. Additional contacts with Asn951 further reinforced binding stability. The ligand extended across the active gorge and interacted with surrounding residues such as Asp884, Asp950, Met804, Glu880, Thr886, Pro810, and Lys883, forming a dense interaction network. These interactions comprised π–π stacking (*T*‐shaped), π‐alkyl, π‐sulfur, π‐amide stacking, alkyl interactions, and van der Waals contacts, collectively contributing to a tightly packed and energetically favorable complex (Figure 7 and Table 1). Minor contributions from carbon‐hydrogen bonding (Ile881) and hydrophobic interactions involving Ile879 and Ile963 further stabilized the ligand within the pocket.

**Table 1:** Docking energy and Interaction of the test drug Hesperidin with the active residues of PI3K.

	CDOCKER interaction energy	Types of interactions	Key residues
Hesperidin	−69.1339	Conventional Hydrogen bond	Val882, Asp964, Tyr867, Asp841, Lys833
		Carbon Hydrogen bond	Ile881
		Alkyl	Ile879
		Pi‐alkyl	Ile963
		Van der Waals	Ile881, Lys883

**Figure 7:**
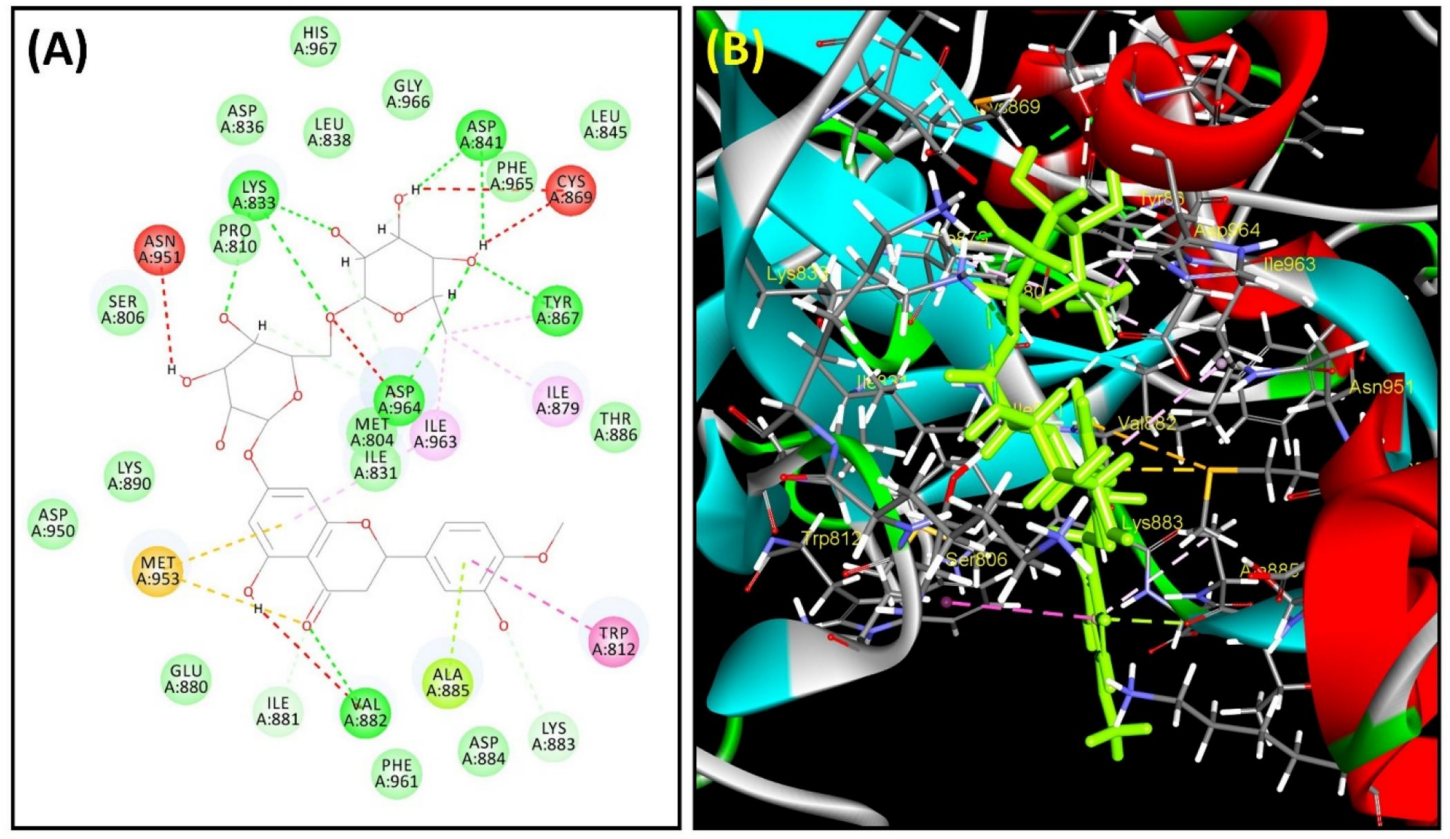
(A) 2D Interaction of HESP with the active residues of PI3Kreceptor. (B) 3D Interaction of HESP with the active residues of PI3K receptor. HESP, hesperidin; PDB, Protein Data Bank; PI3K, phosphoinositide 3‐kinase.

MD simulations over a 100 ns trajectory provided additional insight into the stability of the HESP‐PI3K complex. The protein backbone RMSD displayed an initial equilibration phase followed by stabilization, with fluctuations confined within ~2.0–2.5 Å, indicating preservation of structural integrity throughout the simulation. (Figure 8). The ligand remained consistently positioned within the binding pocket, with no significant displacement observed. Detailed interaction profiling during the simulation demonstrated sustained contacts with several key residues. Notably, hydrophobic interactions with Ile881, Trp812, and Ala805, electrostatic association with Lys833, and polar interactions with Thr887 were maintained throughout the simulation. These persistent interactions were supported by interaction fraction analysis, confirming the stability of both hydrogen bonding and hydrophobic contacts throughout the trajectory (Figure 8).

**Figure 8:**
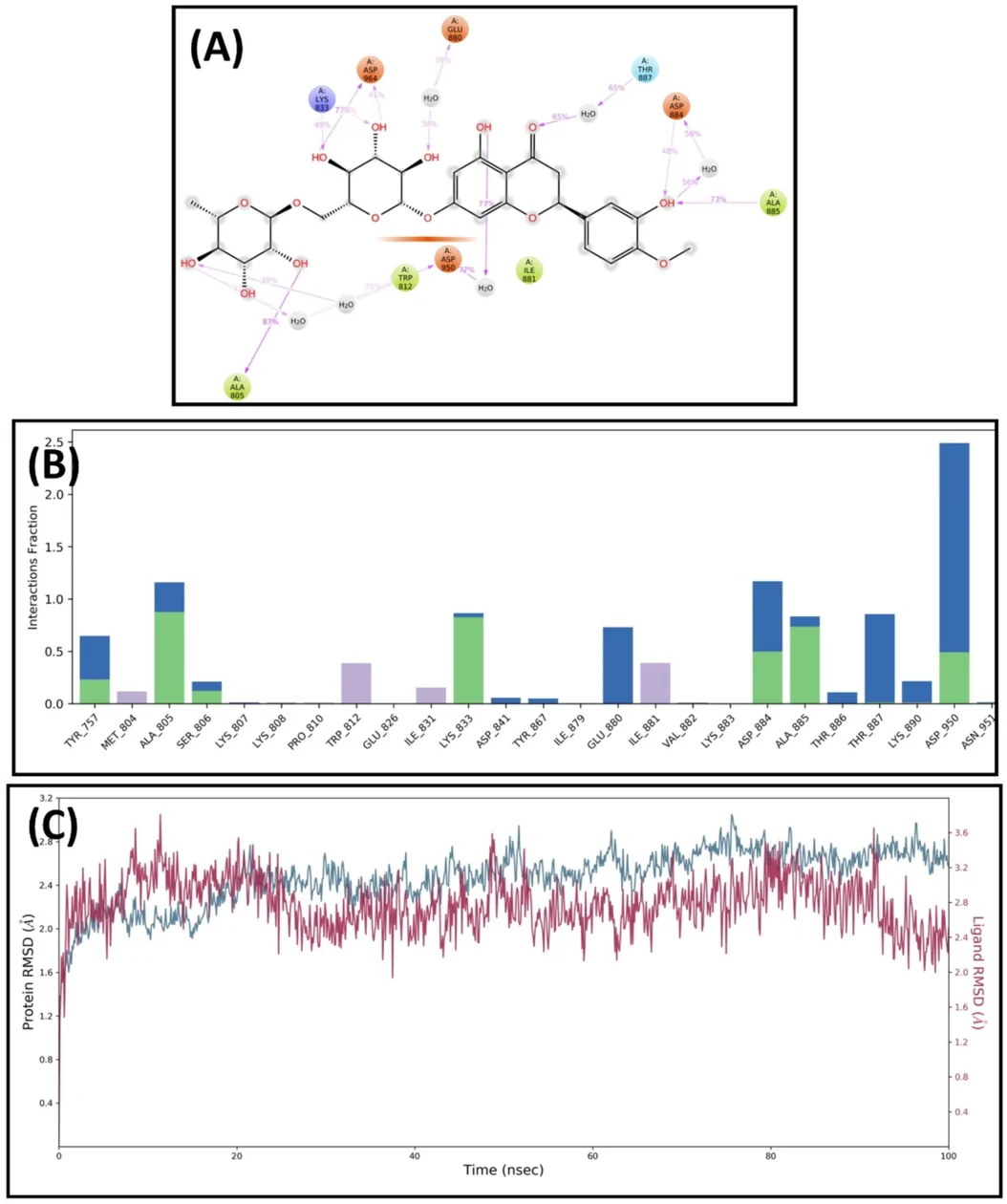
Post‐MD interactions of hesperidin with PI3K. (A) Protein interactions fractions with the ligand (hesperidin) plot throughout the simulation; (B) Protein‐ligand contacts plot of hesperidin with PI3K; (C) RMSD trajectory plot for hesperidin with PI3K. HESP, hesperidin; PDB, Protein Data Bank; PI3K, phosphoinositide 3‐kinase.

## 4 Discussion

The present study was undertaken to evaluate the antidepressant‐like effects of HESP in a CUMS model and to investigate the possible contribution of PI3K signaling to these effects. The major findings demonstrate that CUMS produced a coordinated pattern of behavioral, neuroendocrine, oxidative, inflammatory, and monoaminergic disturbances, while HESP attenuated these abnormalities. Importantly, pharmacological inhibition of PI3K with LY294002 substantially reduced the beneficial effects of HESP across behavioral and biochemical endpoints. Molecular docking and MD simulations further showed favorable and stable interaction of HESP with PI3K. Collectively, these findings support the hypothesis that PI3K signaling may contribute to the antidepressant‐like and neuroprotective actions of HESP under chronic stress conditions, although direct molecular activation of the pathway was not measured.

Chronic stress is a major precipitating factor in depression and can produce anhedonia, reduced motivation, psychomotor impairment, and altered exploratory behavior [48]. The CUMS paradigm is among the most extensively validated experimental models for depression because it reproduces several behavioral, neurochemical, endocrine, and molecular alterations that closely resemble human depressive pathology [49]. In experimental animals, these depressive‐like symptoms can be reliably evaluated using established behavioral paradigms such as the SPT and the OFT [50, 51]. In the present study, CUMS significantly reduced sucrose preference and locomotor activity in the OFT, confirming the development of stress‐associated depressive‐like behavioral dysfunction. These findings are consistent with previous observations that chronic unpredictable stress reduces hedonic behavior and exploratory activity in rodents [52, 53]. Treatment with HESP significantly ameliorated the behavioral deficits induced by CUMS. HESP administration restored sucrose preference and improved locomotor and exploratory activity in the OFT, indicating marked antidepressant‐like activity. These findings are consistent with earlier studies reporting the beneficial effects of HESP in stress‐induced depressive models and other neurobehavioral paradigms, thereby supporting its ability to improve emotional and motivational dysfunction associated with depression [21, 54]. Importantly, the attenuation of HESP‐induced behavioral improvement by LY294002 provides pharmacological evidence supporting the involvement of PI3K signaling in the observed antidepressant‐like effects of HESP under chronic stress conditions.

Chronic stress is also accompanied by dysregulation of the HPA axis, with excessive glucocorticoid secretion contributing to neuronal and behavioral dysfunction [8]. In rodents, corticosterone is an important indicator of stress‐axis activation, and sustained glucocorticoid exposure can promote oxidative injury, inflammatory signaling, and disturbances in monoaminergic neurotransmission [55, 56, 57]. Consistent with this relationship, CUMS markedly increased corticosterone levels in the present study. HESP significantly reduced this elevation, indicating attenuation of stress‐associated endocrine dysregulation. Similar effects of HESP on stress‐related endocrine abnormalities have been reported previously [22]. Importantly, pre‐administration of the PI3K inhibitor LY294002 attenuated the corticosterone‐lowering effect of HESP, resulting in elevated corticosterone levels relative to HESP treatment alone. This observation suggests that PI3K signaling may contribute to HESP‐mediated regulation of HPA axis activity.

Oxidative imbalance is another important component of chronic stress‐induced neuronal dysfunction. Persistent stress and prolonged glucocorticoid exposure can increase reactive oxygen species generation, promote lipid peroxidation, impair mitochondrial function, and weaken endogenous antioxidant defenses [6, 58, 59]. The PI3K signaling cascade plays an essential role in maintaining antioxidant homeostasis, mitochondrial integrity, and neuronal survival [9, 60]. Activation of PI3K signaling promotes antioxidant defense mechanisms and protects against oxidative injury under chronic stress, whereas impairment of this pathway enhances ROS generation, mitochondrial dysfunction, and cellular vulnerability under stress conditions [15]. Thus, oxidative stress and PI3K signaling dysfunction appear to be closely interconnected pathological events contributing to depressive pathology. In agreement with previous findings, CUMS in the present study increased TBARS and reduced GSH, SOD, and catalase, indicating enhanced lipid peroxidation and compromised antioxidant capacity [61]. HESP significantly improved each of these parameters, supporting its ability to counteract stress‐associated redox imbalance. These observations are consistent with the reported antioxidant and neuroprotective properties of HESP [21, 26]. HESP has also been reported to influence intracellular survival and redox‐related signaling pathways, including PI3K‐associated mechanisms [16, 25]. Importantly, LY294002 substantially diminished the antioxidant effects of HESP, with increased TBARS and reduced GSH, SOD, and catalase compared with HESP alone. These findings suggest that PI3K signaling may contribute to the redox‐regulating effects of HESP.

Neuroinflammation provides another mechanistic link between chronic stress and depressive‐like behavior [62]. Persistent stress can promote inflammatory signaling and increase the production of cytokines such as TNF‐α, IL‐1β, and IL‐6, while NF‐κB represents an important transcriptional regulator of inflammatory responses [63, 64, 65, 66]. Increasing evidence suggests that impaired signaling plays a major role in stress‐induced inflammatory dysregulation [67]. PI3K‐related signaling is involved in neuronal survival and cellular homeostasis, and altered PI3K/Akt signaling has been associated with inflammatory and psychiatric disorders [11, 68]. Consistent with this literature, CUMS increased brain TNF‐α, IL‐1β, IL‐6, and NF‐κB levels in the present study. HESP significantly reduced these inflammatory alterations at both doses, supporting its anti‐inflammatory activity under chronic stress. Similar anti‐inflammatory effects of HESP have been reported in experimental neurological models [16, 25]. Importantly, pre‐administration of the PI3K inhibitor LY294002 attenuated the protective effects of HESP and resulted in elevated cytokine and NF‐κB levels compared to HESP treatment alone. These findings suggest a possible involvement of PI3K signaling in the anti‐inflammatory and neuroprotective actions of HESP under chronic stress conditions.

Chronic stress also disrupts monoaminergic neurotransmission, particularly serotonin and dopamine, which are central to mood regulation, reward processing, motivation, and emotional adaptation [69]. Stress can alter the synthesis, release, turnover, and synaptic availability of these neurotransmitters, contributing to anhedonia and reduced motivation [70, 71]. Serotonin plays a crucial role in the regulation of mood, emotional stability, and stress adaptation, whereas dopamine is critically involved in reward processing, motivation, psychomotor activity, and reinforcement behavior [72, 73, 74]. Interestingly, increasing evidence suggests that stress‐induced impairment of PI3K‐related signaling may also contribute significantly to monoaminergic dysfunction [75]. Experimental studies have shown that suppression of PI3K activity can impair serotonergic and dopaminergic neuronal function, whereas restoration of PI3K signaling improves monoaminergic transmission and behavioral outcomes in depressive disorders [12, 68]. In the present study, CUMS significantly reduced brain serotonin and dopamine levels, consistent with previous observations in experimental models of depression. HESP treatment increased both neurotransmitters, suggesting improvement of stress‐associated monoaminergic dysfunction. These findings are compatible with earlier reports describing the ability of HESP to modulate monoaminergic neurotransmission and depressive‐like behavior [21, 23, 26]. The beneficial effects of HESP on monoamines were substantially attenuated by LY294002. Given the established role of monoaminergic neurotransmission in mood regulation and stress responsiveness, this observation suggests that PI3K signaling may contribute to the neurochemical effects of HESP

The PI3K/Akt signaling pathway represents a critical intracellular survival cascade involved in the regulation of neuronal plasticity, stress adaptation, neurotransmission, redox homeostasis, and cellular integrity under physiological conditions [9, 76]. Activation of PI3K leads to phosphorylation of Akt, which subsequently regulates multiple downstream mediators associated with mitochondrial function, glucose metabolism, protein synthesis, synaptic transmission, and anti‐apoptotic signaling [9]. Through modulation of these downstream targets, PI3K signaling maintains neuronal adaptability and protects neural cells against stress‐induced cellular dysfunction. Conversely, impairment of PI3K signaling during chronic stress conditions has been associated with disruption of neuronal homeostasis and progression of depressive pathology [77]. Mechanistically, suppression of PI3K signaling promotes mitochondrial dysfunction and compromises electron transport chain efficiency, thereby increasing leakage of reactive oxygen species and enhancing oxidative damage to membrane lipids and cellular proteins. Such alterations facilitate lipid peroxidation and oxidative neuronal injury, ultimately contributing to synaptic dysfunction and impaired neuronal communication [78, 79]. In parallel, PI3K signaling also regulates cellular inflammatory balance through modulation of stress‐responsive transcriptional pathways. Apart from redox and inflammatory regulation, PI3K signaling is critically involved in the maintenance of monoaminergic neurotransmission [80]. Akt‐dependent signaling modulates neurotransmitter synthesis, vesicular storage, synaptic release, transporter activity, and receptor‐associated neuronal responsiveness. Impairment of PI3K signaling has therefore been associated with depletion of serotonin and dopamine transmission, contributing to anhedonia, emotional dysfunction, motivational deficits, and behavioral despair observed during depression [81]. Furthermore, PI3K signaling also participates in the regulation of stress‐associated endocrine responses by influencing cellular adaptation to glucocorticoid exposure and maintaining neuroendocrine stability under chronic stress conditions [11]. Thus, dysfunction of PI3K signaling creates a pathological environment characterized by oxidative imbalance, inflammatory activation, monoaminergic disturbances, endocrine dysregulation, and impaired neuronal adaptability, all of which collectively contribute to depressive‐like behavior [11].

Collectively, the findings indicate that modulation of PI3K signaling may be associated with the neuroprotective and antidepressant‐like actions of HESP against chronic stress‐induced depressive pathology. The ability of HESP to improve behavioral abnormalities, restore monoaminergic neurotransmission, attenuate oxidative damage, suppress inflammatory mediators, and normalize corticosterone levels suggests coordinated regulation of multiple pathological processes linked with PI3K signaling. More importantly, pharmacological inhibition of PI3K using LY294002 markedly attenuated the beneficial effects of HESP, supporting the involvement of PI3K signaling in mediating, at least in part, its neuroprotective and antidepressant‐like effects. Collectively, these findings suggest that PI3K signaling may contribute to the regulation of oxidative, inflammatory, endocrine, and monoaminergic alterations associated with chronic stress.

In addition to the in vivo findings, molecular docking and MD analyses were performed to further explore the possible interaction of HESP with the PI3K protein and to provide mechanistic support for the observed pharmacological effects. Molecular docking and MD simulations are useful for exploring ligand binding orientation and complex stability [82, 83]. Molecular docking showed favorable accommodation of HESP within the catalytic pocket of PI3K, with a CDOCKER interaction energy of −69.1339 kcal/mol and multiple hydrogen‐bonding and hydrophobic contacts involving residues including Asp841, Lys833, Tyr867, Asp964, and Val882. These interactions suggest structural compatibility between HESP and the PI3K binding site. A 100‐ns MD simulation further demonstrated relative stability of the HESP–PI3K complex, with sustained interactions involving residues such as Ile881, Trp812, Ala805, Lys833, and Thr887 and limited protein backbone fluctuation. The presence of multiple hydrogen bonding and hydrophobic interactions suggests strong molecular complementarity between HESP and the PI3K binding site, thereby supporting the possibility of direct modulation of PI3K signaling by HESP.

Collectively, the molecular docking and MD simulation findings provide additional mechanistic evidence supporting the involvement of PI3K signaling in the neuroprotective and antidepressant‐like actions of HESP. The stable interaction profile of HESP with functionally important residues of PI3K, along with sustained complex stability during simulation analysis, supports the possibility that HESP may modulate PI3K‐mediated intracellular signaling pathways associated with oxidative balance, inflammatory regulation, monoaminergic neurotransmission, neuronal survival, and stress adaptation. These in silico observations, therefore. complement the in vivo findings and further strengthen the hypothesis that PI3K signaling represents a key mechanistic target underlying the antidepressant‐like effects of HESP under chronic stress conditions.

Although the present study demonstrated significant antidepressant‐like and neuroprotective effects of HESP against chronic stress‐induced depressive pathology, certain limitations should be acknowledged. Although the involvement of PI3K was supported by pharmacological inhibition using LY294002 and by molecular docking and MD analyses, changes in PI3K/Akt pathway activation, such as PI3K, phosphorylated Akt (p‐Akt), or downstream signaling proteins, were not directly assessed. Therefore, future studies employing western blotting, immunohistochemistry, or RT‐PCR approaches are required to further validate the precise molecular involvement of PI3K signaling in mediating the effects of HESP. In addition, the present investigation mainly focused on oxidative stress, inflammatory mediators, endocrine dysfunction, and monoaminergic alterations. Histopathological evaluation of brain tissues was also not performed, which could have provided additional structural evidence regarding neuronal damage and neuroprotection under chronic stress conditions. Therefore, future studies incorporating detailed histopathological and immunohistochemical analyses may further strengthen the understanding of cellular and morphological alterations associated with HESP treatment. Moreover, other important mechanisms associated with depression, including neurogenesis, synaptic plasticity, mitochondrial bioenergetics, apoptotic signaling, and neurotrophic pathways such as BDNF/CREB signaling, remain to be explored in relation to HESP‐mediated neuroprotection. Since PI3K signaling interacts closely with multiple intracellular pathways involved in neuronal survival and stress adaptation, future mechanistic studies may provide deeper insight into the broader molecular network underlying the antidepressant‐like effects of HESP. Despite these limitations, the present findings provide pharmacological and computational evidence suggesting that PI3K signaling may contribute to the antidepressant‐like and neuroprotective effects of HESP, warranting further molecular investigation of the PI3K/Akt pathway.

## Author Contributions

**Mimansa Kandhwal:** data curation. **Amarjot Kaur Grewal:** supervision, writing – original draft. **Komal Thapa:** investigation. **Varinder Singh:** writing – review and editing. **Manjinder Singh:** methodology. **Tanveer Singh:** formal analysis. **Thakur Gurjeet Singh:** project administration, resources. **Sheikh F. Ahmad:** funding acquisition. **Gamaleldin I. Harisa:** funding acquisition, resources.

## Conflicts of Interest

The authors declare no conflicts of interest.

## Data Availability

The data that support the findings of this study are available from the corresponding author upon reasonable request. Data will be made available on request.
